# Supplementary material for: The Effect of the Immunization Schedule and Antibody Levels (Anti-S) on the Risk of SARS-CoV-2 Infection in a Large Cohort of Healthcare Workers in Northern Italy
Source: Vaccines (Basel). 2023 Mar 28;11(4):746. doi: 10.3390/vaccines11040746 (PMC10144408; doi:10.3390/vaccines11040746)
Supplement: Supplementary file 1 [file vaccines-11-00746-s001.zip › vaccines-2267751-supplementary.pdf]

vaccines

MDPI

Supplementary material

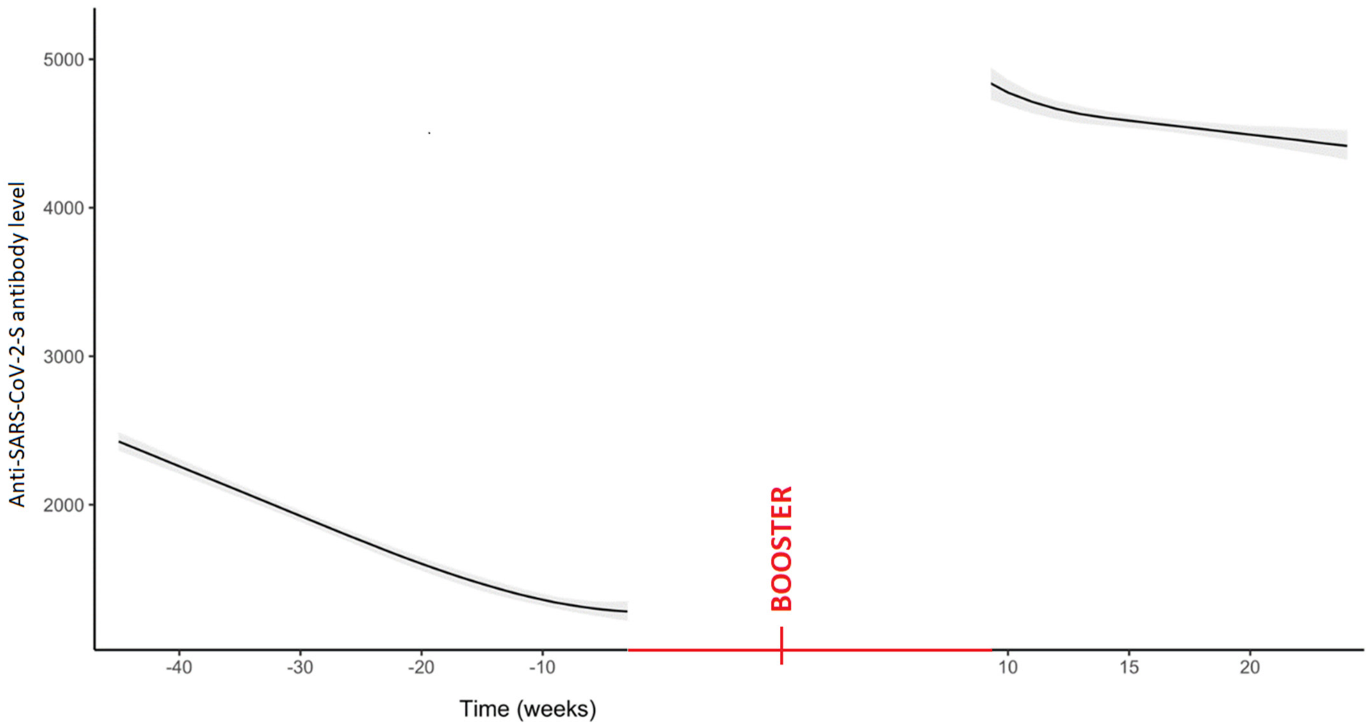

Figure S1. Overall trend of the anti-S antibody levels (U/mL) over time. Curves were obtained from the predictions of the bootstrapped piecewise linear mixed model. The booster injection date was set as the reference for time. The gap between the two periods (before and after the booster) is due to the absence of an anti-S serological assay performed during the interval between T_3 and T_4, when the booster dose was administered.

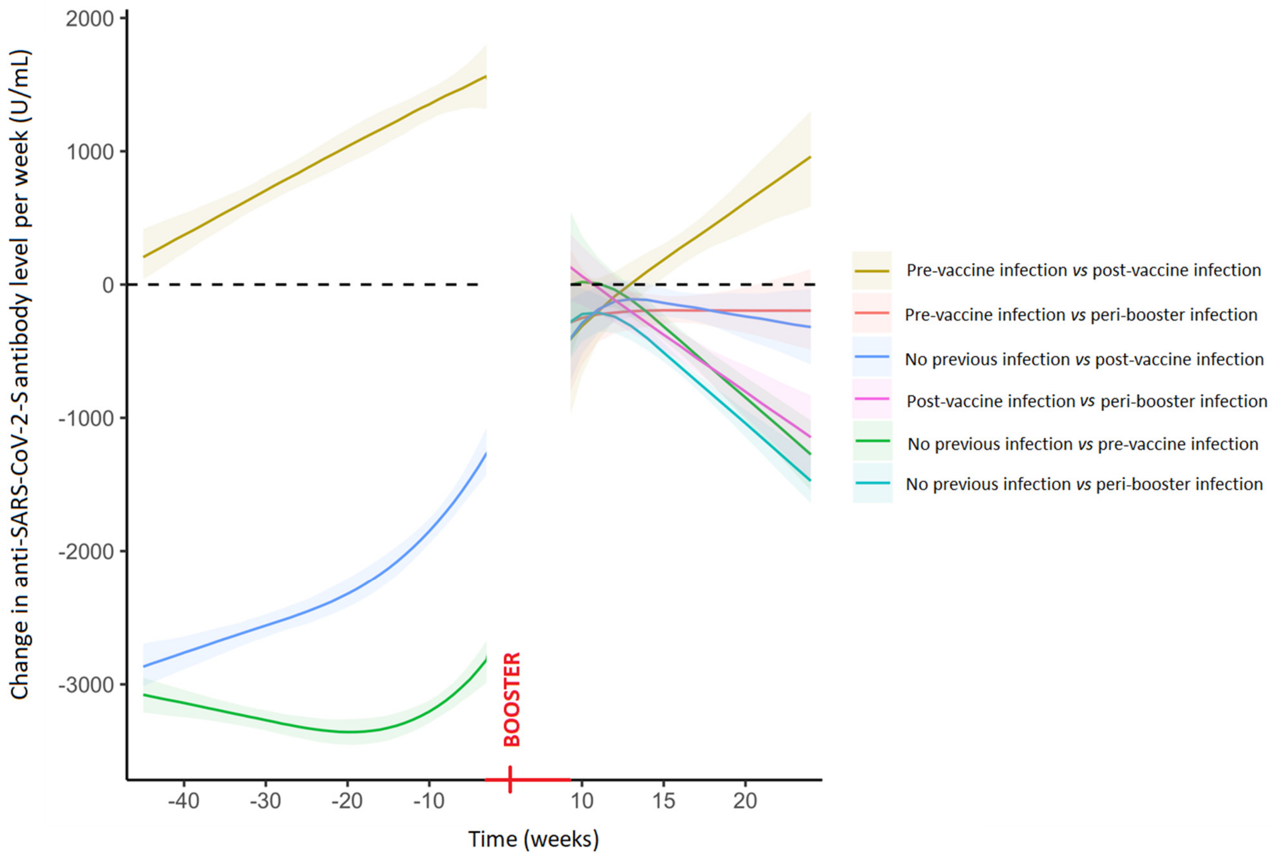

Figure S2. Difference of the anti-S antibody levels (U/mL) over time comparing the different groups identified by the occurrence of previous SARS-CoV-2 infection. Curves were obtained from the predictions of the bootstrapped piecewise linear mixed model adjusted by age and gender. The day of the booster injection was set as the reference for time. The gap between the two periods (before and after the booster) is due to the absence of an anti-S serological assay performed during the interval between T_3 and T_4, when the booster dose was administered. Due to the infections occurred in such interval the no previous infection group was split in two, originating the peri-booster infection group. Comparing curves referring to the peri-booster infection group can be only seen on the right part of the figure.

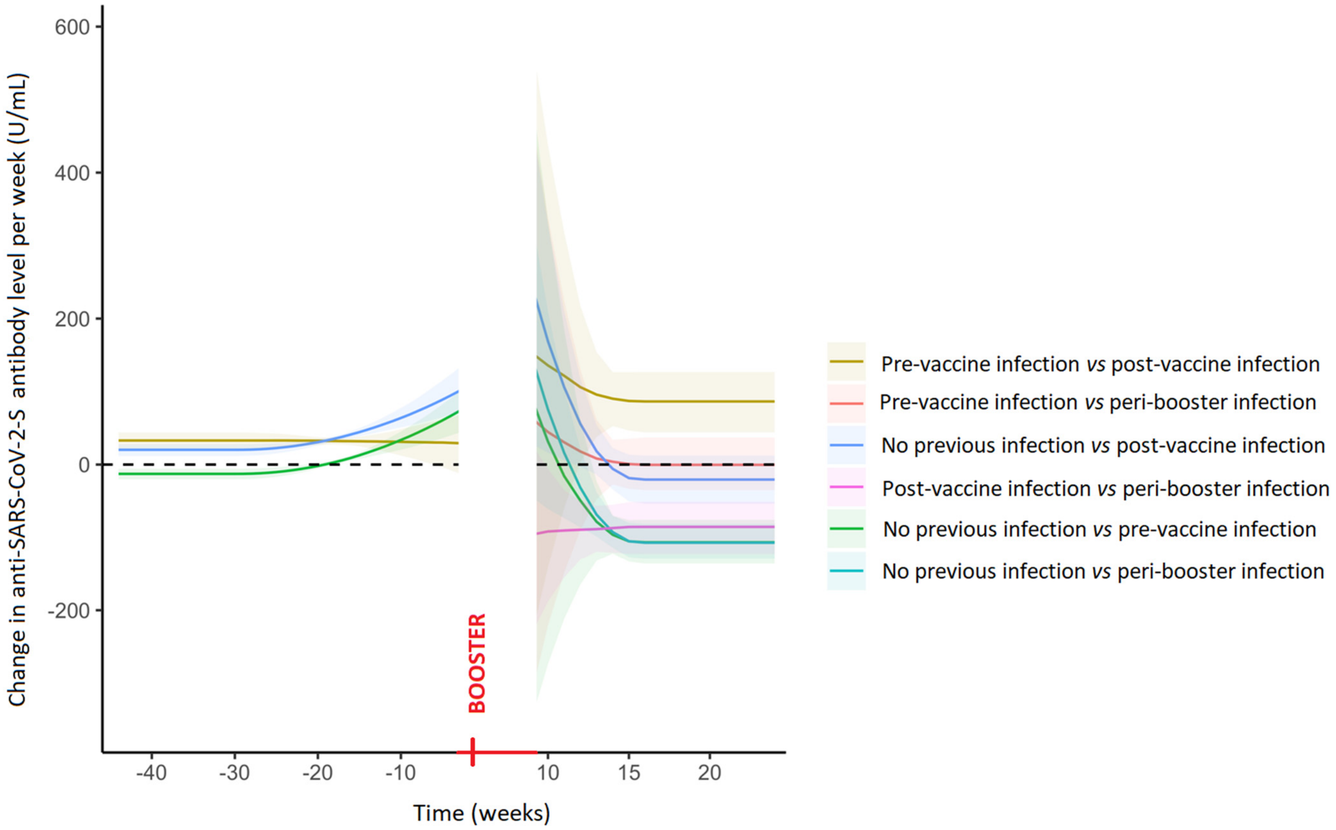

Figure S3. Difference of the gradient of the anti-S antibody levels (U/mL) over time comparing the different groups identified by the occurrence of previous SARS-CoV-2 infection. Curves were obtained from the gradient of the predictions of the bootstrapped piecewise linear mixed model adjusted by age and gender. The day of the booster injection was set as the reference for time. The gap between the two periods (before and after the booster) is due to the absence of an anti-S serological assay performed during the interval between T_3 and T_4, when the booster dose was administered. Due to the infections occurred in such interval the no previous infection group was split in two, originating the peri-booster infection group. Comparing curves referring to the peri-booster infection group can be only seen on the right part of the figure.

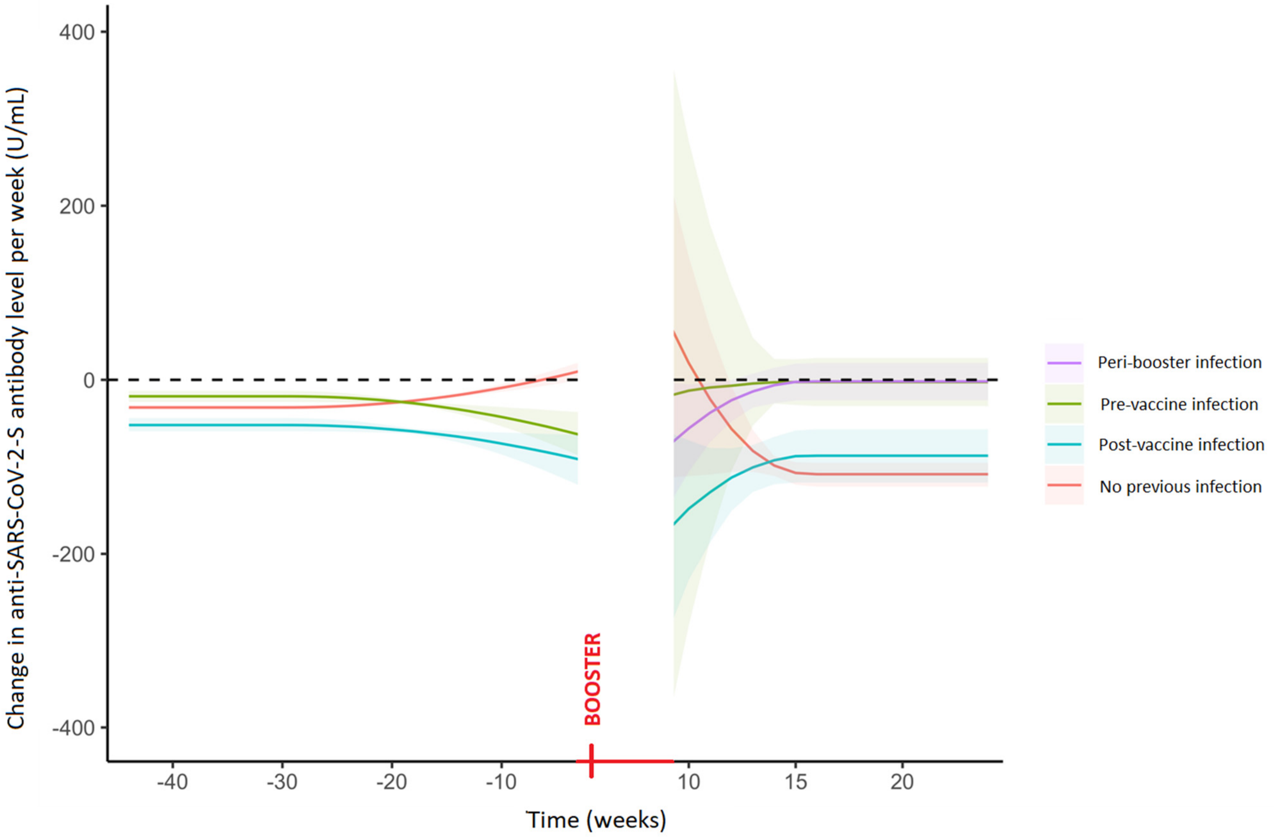

Figure S4. Gradient of the anti-S antibody levels (U/mL) over time stratified by the occurrence of previous SARS-CoV-2 infection. Curves were obtained from the gradient of the predictions of the bootstrapped piecewise linear mixed model adjusted by age and gender. The day of the booster injection was set as the reference for time. The gap between the two periods (before and after the booster) is due to the absence of an anti-S serological assay performed during the interval between T_3 and T_4, when the booster dose was administered. Due to the infections occurred in such interval the no previous infection group was split in two, originating the peri-booster infection group, which curve can be only seen on the right part of the figure.

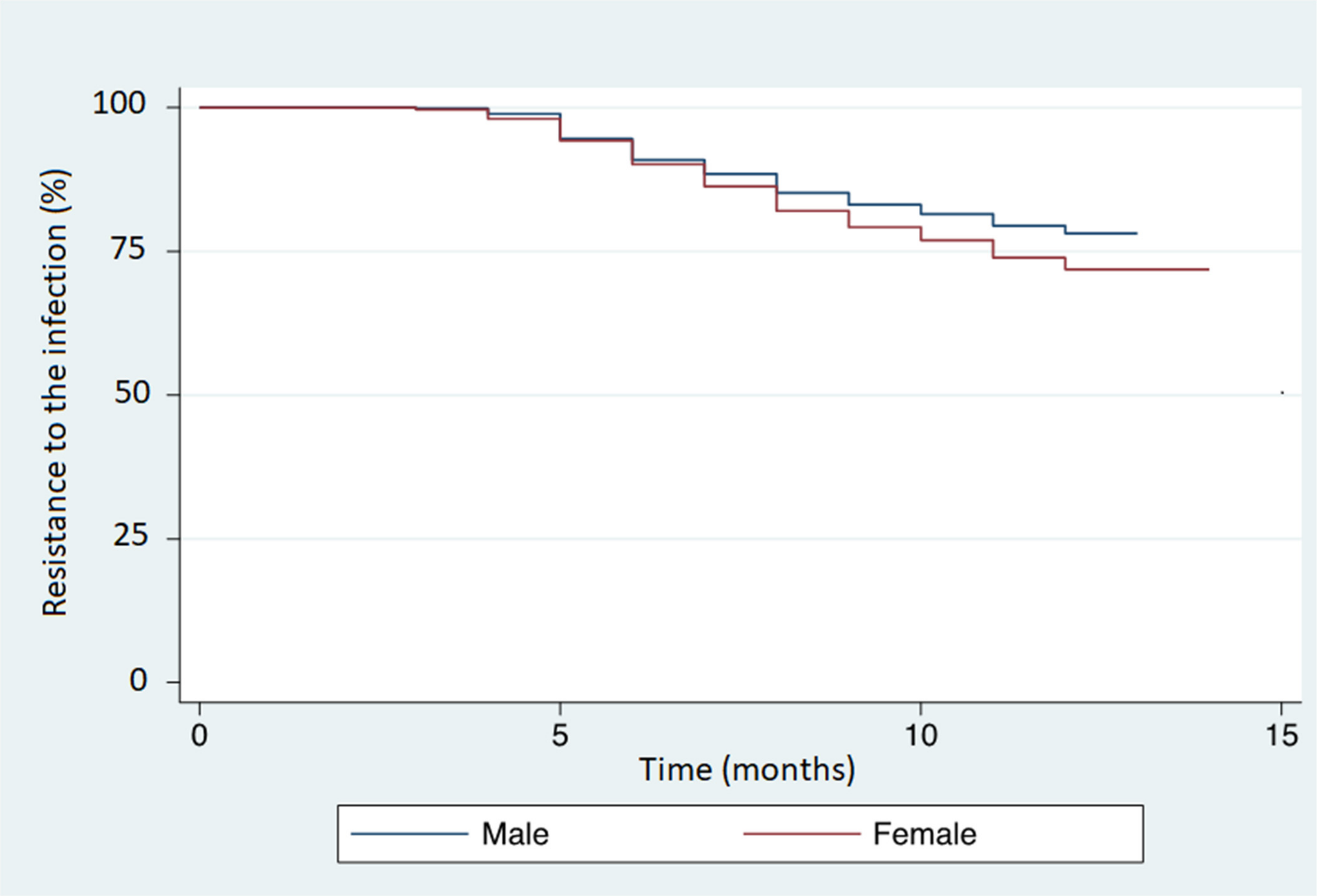

Figure S5. Kaplan-Meier curves measuring time without infection obtained according to gender. The booster injection date was considered a reference for the time axis.

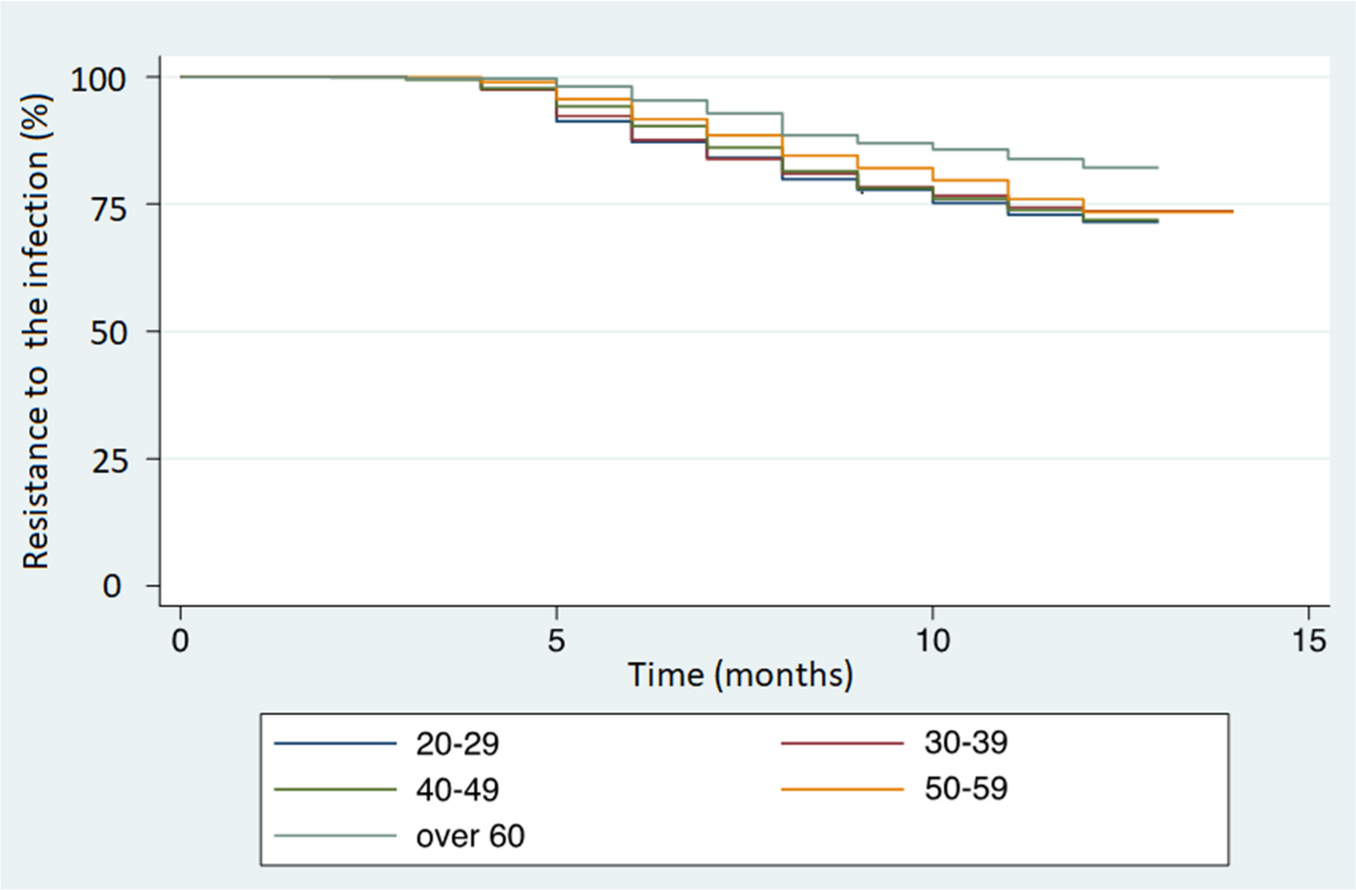

Figure S6. Kaplan-Meier curves measuring time without infection obtained according to considered age groups. The booster injection date was considered a reference for the time axis.

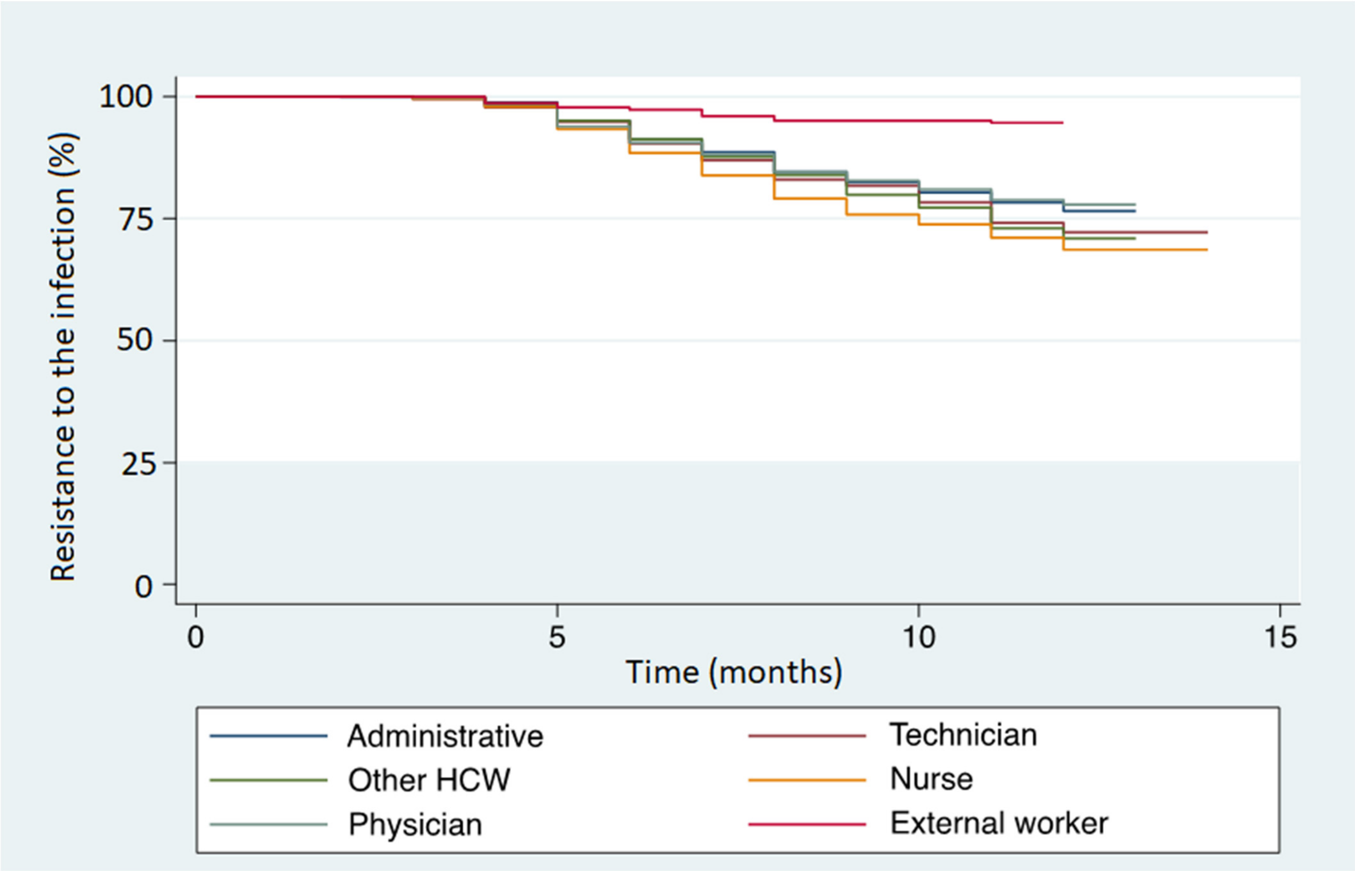

Figure S7. Kaplan-Meier curves measuring time without infection obtained according to considered job titles. The booster injection date was considered a reference for the time axis.

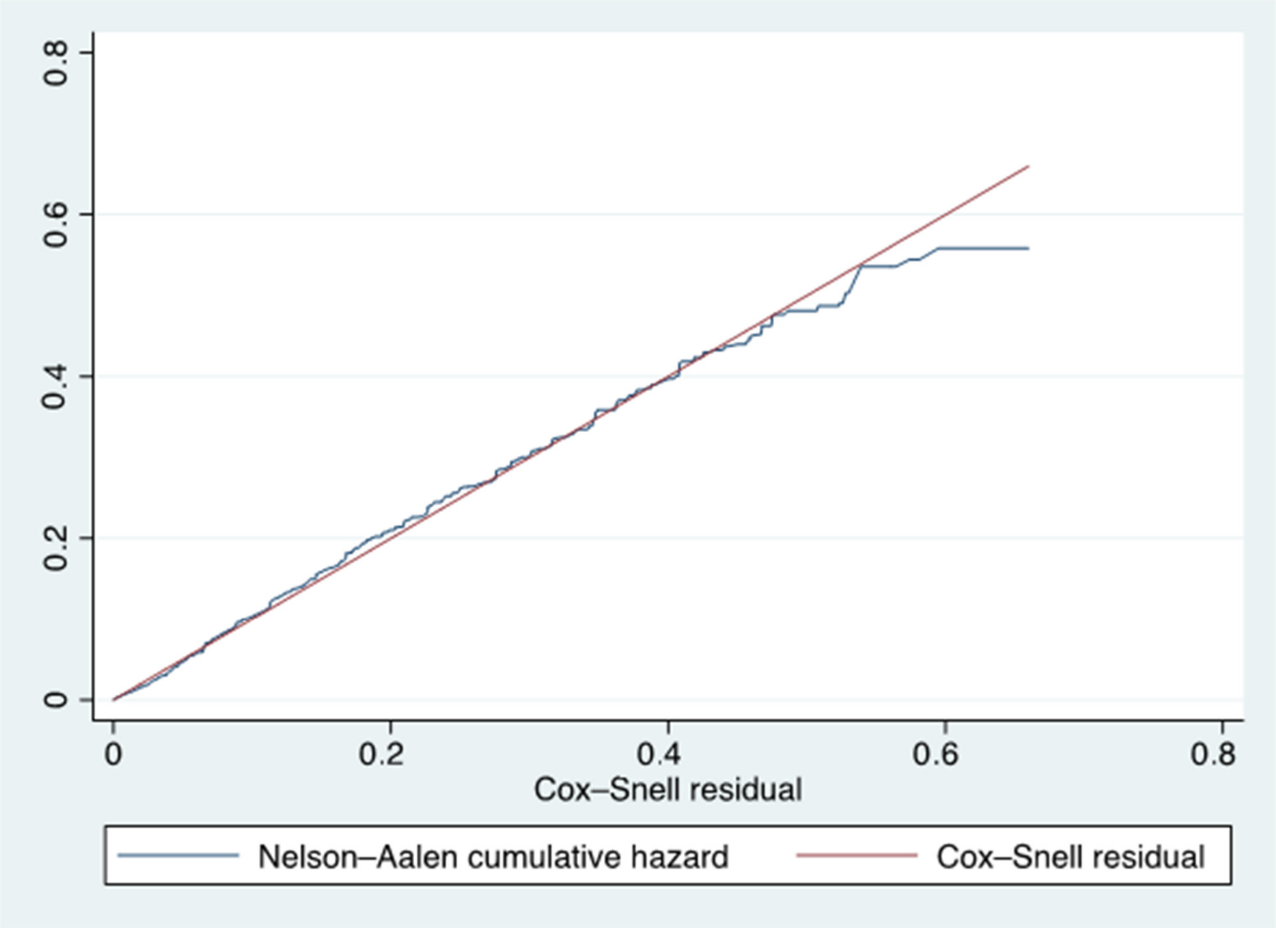

Figure S8. The goodness of fit of the model is verified by Cox-Snell residuals. Interpretation: the model fits the data well if the cumulative hazard function approximately follows the straight line.

Table S1. Survival data description. A failure event is defined as COVID-19 infection after the booster dose. Time 0 is the date of the booster dose administration. The total number of subjects enrolled in the study was 4824. Each subject is followed for a variable number of days, starting from the date of booster administration. A total of 1250 subjects were infected (event = 1) during the follow-up time. All the other subjects are censored (event = 0). The longest follow-up time recorded was 14 months.

**Total observations**	**4824**	
**Exclusions**	0	
**Failure**	event = 1	
**Analysis time**	months	
**Failures**	1250	
	**At risk from t =**	0
	**Earliest observed entry t =**	0
	**Last observed exit =**	14

		**Per subject**		
**Category**	**Mean**	**Min**	**Median**	**Max**
Entry time (first)	0	0	0	0
Exit time (final)	10.7	2	11.5	14
Time at risk	10.7	2	11.5	14

Table S2. Analysis of time (months) variable for infected subjects. The table provides some descriptive statistics for the time variable, only related to the subjects for which infection is recorded. On average, infection was recorded between 7 and 8 months after the booster dose.

Analysis time when record ends				
	**Percentiles**	**Smallest**		
1%	3	2		
5%	4	3	**Obs**	1,250
10%	5	3		
25%	6	3	**Mean**	7.59
50%	7	**Largest**	**SD**	2.30
75%	9	12		
90%	11	12	**Variance**	5.28
95%	11	12	**Skewness**	0.21
99%	12	12	**Kurtosis**	2.07

Table S3. The log-rank test was used to test the null hypothesis of no difference in survival between subjects with different immunization profiles. A rejection of the null hypothesis for the log-rank test is also obtained when comparing groups of subjects with different genders, ages or job titles.

Equality of survivor functions ǀ Log-rank test		
**Immunization profile**	**Observed events**	**Expected events**
Prevaccine infection (< T0)	195	262.97
Post-vaccine infection (T0-T3)	22	19.28
Peri-booster infection (> T3)	50	261.32
No natural infection	983	706.43
Total	1250	1250
	chi2 (3) =	308.77
	p value =	0.0000
